# Induced pluripotent stem cells modulate the Wnt pathway in the bleomycin-induced model of idiopathic pulmonary fibrosis

**DOI:** 10.1186/s13287-023-03581-4

**Published:** 2023-11-28

**Authors:** Paria Bayati, Marjan Taherian, Mansoureh Soleimani, Hamid Farajifard, Nazanin Mojtabavi

**Affiliations:** 1 Cellular and Molecular Research Center, Iran University of Medical Sciences, Tehran, Iran; 2 Immunology Research Center, Department of Immunology, School of Medicine, Iran University of Medical Sciences, Tehran, Iran; 3 Pediatric Cell and Gene Therapy Research Center, Tehran University of Medical Sciences, Tehran, Iran

**Keywords:** WNT, b-Catenin, BMP4, DKK1, IPF, IPSC, Fibrosis

## Abstract

**Background:**

The Wnt signaling pathway has been implicated in the pathogenesis of fibrotic disorders and malignancies. Hence, we aimed to assess the potential of the induced pluripotent stem cells (IPS) in modulating the expression of the cardinal genes of the Wnt pathway in a mouse model of idiopathic pulmonary fibrosis (IPF).

**Methods:**

C57Bl/6 mice were randomly divided into three groups of Control, Bleomycin (BLM), and BLM + IPS; the BLM mice received intratracheal instillation of bleomycin, BLM + IPS mice received tail vein injection of IPS cells 48 h post instillation of the BLM; The Control group received Phosphate-buffered saline instead. After 3 weeks, the mice were sacrificed and Histologic assessments including hydroxy proline assay, Hematoxylin and Eosin, and Masson-trichrome staining were performed. The expression of the genes for Wnt, β-Catenin, Lef, Dkk1, and Bmp4 was assessed utilizing specific primers and SYBR green master mix.

**Results:**

Histologic assessments revealed that the fibrotic lesions and inflammation were significantly alleviated in the BLM + IPS group. Besides, the gene expression analyses demonstrated the upregulation of Wnt, β-Catenin, and LEF along with the significant downregulation of the Bmp4 and DKK1 in response to bleomycin treatment; subsequently, it was found that the treatment of the IPF mice with IPS cells results in the downregulation of the Wnt, β-Catenin, and Lef, as well as upregulation of the Dkk1, but not the Bmp4 gene (*P* values < 0.05).

**Conclusion:**

The current study highlights the therapeutic potential of the IPS cells on the IPF mouse model in terms of regulating the aberrant expression of the factors contributing to the Wnt signaling pathway.

## Introduction

Idiopathic pulmonary fibrosis (IPF) is a chronic and progressive lung disease that affects millions of people worldwide and has a poor prognosis, with a median survival of 3–5 years after diagnosis [1, 2]. IPF is characterized by the accumulation of fibroblasts and extracellular matrix in the alveolar space, leading to impaired gas exchange and respiratory failure [3]. The etiology and pathogenesis of IPF are not fully understood, but several factors have been implicated, such as genetic predisposition, environmental exposure to dust or pollutants, oxidative stress induced by reactive oxygen species, and aberrant wound healing triggered by repeated micro-injuries [1, 3, 4]. Among the molecular mechanisms involved in the development and progression of IPF, the Wnt signaling pathway has emerged as a key player [5]. The Wnt signaling pathway is a complex network of proteins that regulates various cellular processes, such as proliferation, differentiation, migration, and survival; this pathway consists of several ligands (Wnts), receptors (Frizzleds and LRP5/6), co-receptors (R-spondins and Kremen), intracellular mediators (Disheveled and Axin), and transcriptional effectors (β-catenin and lymphoid enhancer factor [LEF]/T-cell factor [TCF]) [6]. In normal lung development and homeostasis, the Wnt signaling pathway is tightly regulated by a balance between positive and negative modulators; However, in IPF, the Wnt signaling pathway is dysregulated, resulting in the activation of downstream effectors, such as β-catenin and LEF/TCF, that promote fibroblast activation and collagen deposition [5, 7–9]. Moreover, the expression of Wnt inhibitors, such as dickkopf-1 (DKK1), secreted frizzle-related proteins (sFRPs), and Wnt inhibitory factor-1 (WIF-1), is reduced in IPF, further enhancing the fibrotic response [5]. Dysregulated Wnt signaling contributes to pulmonary fibrosis by affecting the behavior and interactions of different cell types involved in the pathogenesis of the disease, such as epithelial cells, fibroblasts, and inflammatory cells; Also, Wnt signaling influences the expression and activity of various pro-fibrotic factors and mediators, such as TGF-β, CTGF, EMT, collagen, and myofibroblasts [7]. Therefore, targeting the Wnt signaling pathway may represent a novel therapeutic strategy for IPF.

BMP4 is a member of the bone morphogenetic protein (BMP) family, which belongs to the transforming growth factor (TGF)-β superfamily. BMP4 can bind to specific receptors on the cell surface and activate downstream effectors, such as Smad1/5/9, to modulate gene expression [10]. BMP4 has been shown to have antifibrotic effects in various models of fibrosis, such as pulmonary fibrosis, liver fibrosis, and renal fibrosis. BMP4 can inhibit the differentiation of fibroblasts into myofibroblasts, which are the main ECM-producing cells in fibrotic tissues. BMP4 can also reduce the expression of ECM components, such as collagen and fibronectin, and promote the degradation of ECM by activating matrix metalloproteinases (MMPs) [10–12]. The interplay between BMP4 and WNT signaling in fibrosis is complex and context-dependent. In some cases, BMP4 and WNT signaling can antagonize each other by modulating the activity or expression of their receptors or downstream effectors. For example, BMP4 can inhibit WNT signaling by inducing the expression of Dkk1. Conversely, WNT signaling can inhibit BMP4 signaling by inducing the expression of Smad6, a negative regulator of BMP receptors. In other cases, BMP4 and WNT signaling can cooperate or synergize with each other by regulating common targets or pathways. For example, BMP4 and WNT signaling can both activate YAP/TAZ, a transcriptional coactivator that can promote myofibroblast differentiation and ECM production [13]. Additionally, BMP4 and WNT signaling can both modulate TGF-β signaling, another major pro-fibrotic pathway that can interact with both BMP4 and WNT signaling [14]. Therefore, the interplay between BMP4 and WNT signaling in fibrosis is dynamic and multifaceted. Understanding how these two pathways interact in different organs and stages of fibrosis may provide new insights into the molecular mechanisms of fibrosis and reveal novel therapeutic targets for antifibrotic interventions. Hence, we aimed to assess the gene expression of BMP4 along with the genes involved in the WNT pathway in the context of bleomycin-induced pulmonary fibrosis and after treatment with the iPSCs.

The treatment of IPF patients poses many unresolved challenges and controversies, such as the optimal timing and criteria for initiating antifibrotic therapy [15]. Another common cause of debate is the choice and combination of antifibrotic agents, such as nintedanib and pirfenidone, for IPF patients [16]. Some studies suggest that they may have additive or synergistic effects, while others show no benefit or increased adverse events when used together [17]. Moreover, the role and efficacy of the available therapies, such as immunomodulators, anti-inflammatory agents, anticoagulants, antioxidants, and antimicrobials, for IPF patients are also among the issues that lack a common agreement [18, 19]. Some studies suggest that they may have beneficial effects, while others show no benefit or harmful effects on survival or quality of life.

Induced pluripotent stem cells (iPSCs) are somatic cells that have been reprogrammed to acquire pluripotency and differentiation potential by introducing specific transcription factors, such as Oct4, Sox2, Klf4, and c-Myc [20]. IPSCs have been widely used for regenerative medicine and disease modeling, as they can generate various cell types of interest [21, 22]. In particular, they have shown promising results in the treatment of lung diseases, such as emphysema, pulmonary hypertension, and IPF [23, 24]. IPSCs can modulate the inflammatory and fibrotic processes in the lung by secreting anti-inflammatory and antifibrotic factors, such as interleukin-10 (IL-10), and vascular endothelial growth factor (VEGF) [24–26]. These factors can inhibit the activation of pro-inflammatory and pro-fibrotic cells, such as macrophages, neutrophils, fibroblasts, and myofibroblasts. Moreover, iPSCs can differentiate into alveolar epithelial cells (AECs) and endothelial cells (ECs), which can restore the damaged alveolar structure and function [23]. However, the effects of iPSCs on the Wnt signaling pathway in IPF have not been extensively investigated.

In this study, we aimed to evaluate the potential of iPSCs in modulating the expression of the key genes of the Wnt signaling pathway in a mouse model of IPF induced by bleomycin; this model is well-established and widely used for studying the pathogenesis and treatment of lung fibrosis which mimics many aspects of human idiopathic pulmonary fibrosis (IPF). Bleomycin is an anticancer drug that causes lung injury and fibrosis by inducing oxidative stress and inflammation. We hypothesized that iPSCs would attenuate the fibrotic response by downregulating the expression of Wnt, β-catenin, and Lef, as well as upregulating the expression of Dkk1 and Bmp4 as well. To test this hypothesis, we performed histological and molecular analyses on lung tissues from control mice, bleomycin-treated mice, and bleomycin-treated mice that received iPSCs via tail vein injection.

## Methods

### Animals and experimental design

Male C57BL/6 mice (8–10 weeks old, 17–20 g, healthy and naïve to any laboratory procedure) were obtained from the Center for Experimental and Comparative Studies, Iran University of Medical Sciences. The mice were housed in a specific pathogen-free facility with a 12-h light/dark cycle and free access to food and water. Mouse developing any sign of infection or other complications due to the experimental procedures were decided to be excluded, although no such complications happened during the study. All animal procedures were performed according to the National Institutes of Health Guide for the Care and Use of Laboratory Animals and approved by the Institutional Animal Care and Use Committee of Iran University of Medical Sciences, 2018 (ethics code: IR.IUMS.REC1399.392). The experimental unit in this study was a single mouse, as each mouse receives various treatments and the total number in each experiment was 21. A total number of 21 mice were randomly divided into three groups: Control (n = 7), BLM (n = 7), and BLM + iPSC (n = 7). The order of treatments and measurements, or animal/cage location, were not systematically biased by any known or unknown factors. Due to ethical and practical constraints, we decided to use the minimum number of animals possible; therefore, we used 7 mice per group in our experiment. The Control group received intratracheal instillation of 50 μl of phosphate-buffered saline (PBS) under anesthesia. For induction of pulmonary fibrosis, the BLM and BLM + iPS groups received intratracheal instillation of 50 μl of bleomycin (5 mg/kg) dissolved in PBS. We anesthetized the mice with ketamine and xylazine before instillation. We used a 24-gauge catheter to directly deliver the bleomycin solution, while the mouse was supported on a diagonal surface by about 60 degrees of rotation. Then, we made a fine incision at the skin above the trachea in the neck area and inserted the catheter into the exposed trachea. Then, we sutured the wound and held the animal in an upright position to support maximum breathing capacity and avoid suffocation. We monitored the mice for signs of pain or distress, such as weight loss, reduced activity, or respiratory distress. Subjects suffering from severe ailment/weight loss were anesthetized using ketamine/xylazine and were sacrificed before the humane endpoint of 3 weeks. We also monitored their weight and food and water intake daily. The BLM + iPSC group received intratracheal instillation of bleomycin as above, followed by tail vein injection of 1 × 10^6^ iPSCs suspended in 200 μl of PBS 48 h later. The control and the BLM group received PBS via the tail vein route instead. Only the principal investigator was aware of the allocations; the histologic and molecular assessments were performed blinded. The mice were sacrificed by cervical dislocation under anesthesia with ketamine and xylazine at 3 weeks after bleomycin administration. The lungs were harvested for histological and molecular analyses. The primary outcome measure in this study was fibrosis onset; for the assessment of inflammation and fibrosis, H&E and Masson-trichrome staining, plus hydroxy proline assay were performed. For assessment of the WNT signaling, the gene expression for Wnt, b-Catenin, Lef1, Dkk1, and, Bmp4 was quantified. We used three sections from each lung lobe (right cranial, right middle, right caudal, left cranial, and left caudal) which were randomly allocated to histological and molecular analyses. We randomly chose ten fields from each section for the assessment of fibrotic lesions and inflammation.

### Generation and characterization of iPSCs

To generate induced pluripotent stem cells (IPSCs) from mouse embryonic fibroblasts (MEFs), we used the following protocol. First, we obtained MEFs from 13-week-old pregnant C57BL/6 mice by physical mincing of the embryos in phosphate-buffered saline (PBS) under sterile conditions. Then, we enzymatically dissociated the minced tissues using a mixture of collagenases, trypsin, and DNAse1 (Thermo Fisher, USA). We cultured the isolated MEFs in Dulbecco's Modified Eagle Medium (DMEM) supplemented with 20% fetal bovine serum (FBS) and 0.1 mM β-mercaptoethanol (passage zero). Then, the MEFs were passaged, and the passage two cells were used for lentiviral transduction. Subsequently, we transduced the MEFs with lentiviral particles carrying either OCT4, SOX2, KLF4, and c-MYC (LV-OSKM) or enhanced green fluorescent protein (LV-eGFP) in the presence of polybrene transfection reagent (Merck, Germany) overnight at a multiplicity of infection (MOI) of 15. We used the separate lentiviral vector (LV-eGFP) in parallel to the LV-OSKM as a control to assess the transduction efficiency and to calculate the MOI. We cultured the transduced cells in DMEM complete-plus medium (Merck, Germany) containing 1% recombinant leukemia inhibitory factor (LIF) (R&D Systems, USA) for about 20 days. During this period, the transformed cells showed the characteristic phenotypes of stem cells and formed raised colonies. We manually picked the colonies and enzymatically dissociated them using Accutase cell detachment solution (Merk, Germany). We confirmed the gene expression profile of the IPSCs by quantitative polymerase chain reaction (qPCR) with specific primers recognizing OCT4, SOX2, and KLF4 genes, which were included in the lentiviral vectors, as well as the NANOG gene, which was absent in the lentiviral particles. Then, we compared the gene expression data with non-transduced MEFs. Then, we expanded the IPSCs to obtain enough cells for treating the bleomycin-induced lung fibrosis (BLF) mice.

### Histological assessment

The lung tissues were fixed in 4% paraformaldehyde, embedded in paraffin, and sectioned at 5 μm thickness. The sections were stained with hematoxylin and eosin (H&E) for general morphology and inflammation assessment, or Masson's trichrome for collagen deposition and fibrosis assessment. The images were captured using a light microscope (Olympus BX51) equipped with a digital camera (Olympus DP72). The histologic assessments were carried on by an independent pathologist who was unaware of the sample identity.

### Hydroxyproline assay

The hydroxyproline content in the lung tissues was measured as an indicator of collagen accumulation, using a colorimetric assay by measuring the color results from the reaction of chloramine-T with hydroxyproline residues of the collagen fibers. Briefly, the complete right lungs were homogenized in distilled water and hydrolyzed in 6 N HCl at 120 °C for 18 h. The hydrolysates were neutralized with NaOH and mixed with chloramine-T solution. After incubation at room temperature for 20 min, the mixture was added with p-dimethyl-amino-benzaldehyde solution and heated at 60 °C for 15 min. Subsequently, the absorbance was measured at 550 nm using a microplate reader. The hydroxyproline concentration was then calculated by comparing the results with a standard curve and was normalized to the tissue weight.

### Real-time PCR

The total RNA was extracted from the lung tissues that were kept frozen in the RNALater solution. After fine-mincing, the samples were lysed with a homogenizer in Trizol lysis buffer (SinaClon, Iran) according to the manufacturer's instructions. RNA isolation was carried out using RNXplus (SinaClon, Iran) according to the manufacturer’s protocol. The RNA concentration and purity were determined by gel electrophoresis and NanoDrop spectrophotometer (Thermo Fisher, USA). The reverse transcription was performed using the RevertAid First Strand cDNA Synthesis Kit (Fermentase, USA) according to the manufacturer's protocol. Eventually, the gene expression of Wnt, β-Catenin, Lef, Dkk1, and Bmp4 was assessed utilizing specific primers and the RotorGeneQ real-time PCR instrument (Qiagen, Germany) with SYBR green master mix (Amplicon, Denmark). The primer sequences are listed in Table 1. The relative expression of each gene was calculated by the 2 − ΔΔCT method using mouse Gapdh as an internal control.

**Table 1:** Primer sequences for real-time PCR

Gene name	Sequence (5′–3′)
Lef1 Forward primer	AGAGTGCAGCTATCAACCAGATCC
Lef1 Reverse primer	GCTGTCTCTCTTTCCGTGCTAGTTC
Wnt Forward primer	CCTCATGAACCTTCACAACAACGAG
Wnt Reverse primer	CATTTGCACTCTTGGCGCATC
B-catenin Forward primer	GCCACAGGATTACAAGAAGCGG
B-catenin Reverse primer	GGCACCAATGTCCAGTCCAAG
Dkk1 Forward primer	CCCAGAAGAACCACACTGACTTC
Dkk1 Reverse primer	ATGATCGGAGGCAGACGGAG
Bmp4 Forward primer	ATTGTGCAGACCCTAGTCAACTCT
Bmp4 Reverse primer	AGGTACAACATGGAAATGGCACTC

### Statistical analysis

The data are presented as mean ± standard deviation (SD). The differences among groups were analyzed by one-way analysis of variance (ANOVA) followed by Tukey's post hoc test using GraphPad Prism 8 software. A *p* value < 0.05 was considered statistically significant.

## Results

In the current study, we established a mouse model of idiopathic pulmonary fibrosis (IPF) by intratracheal administration of bleomycin, which caused inflammation and fibrosis in the lung tissue. We confirmed the development and progression of IPF by histological analyses and hydroxyproline assay, which showed increased alveolar damage, collagen deposition, and fibrotic foci in the lungs of bleomycin-treated mice compared to the control mice. We also demonstrated that induced pluripotent stem cells (iPSCs) could ameliorate the histological features and modulate the aberrant expression of Wnt-related genes in the bleomycin-induced IPF model.

### Histological assessment of lung fibrosis

**Fig. 1:**
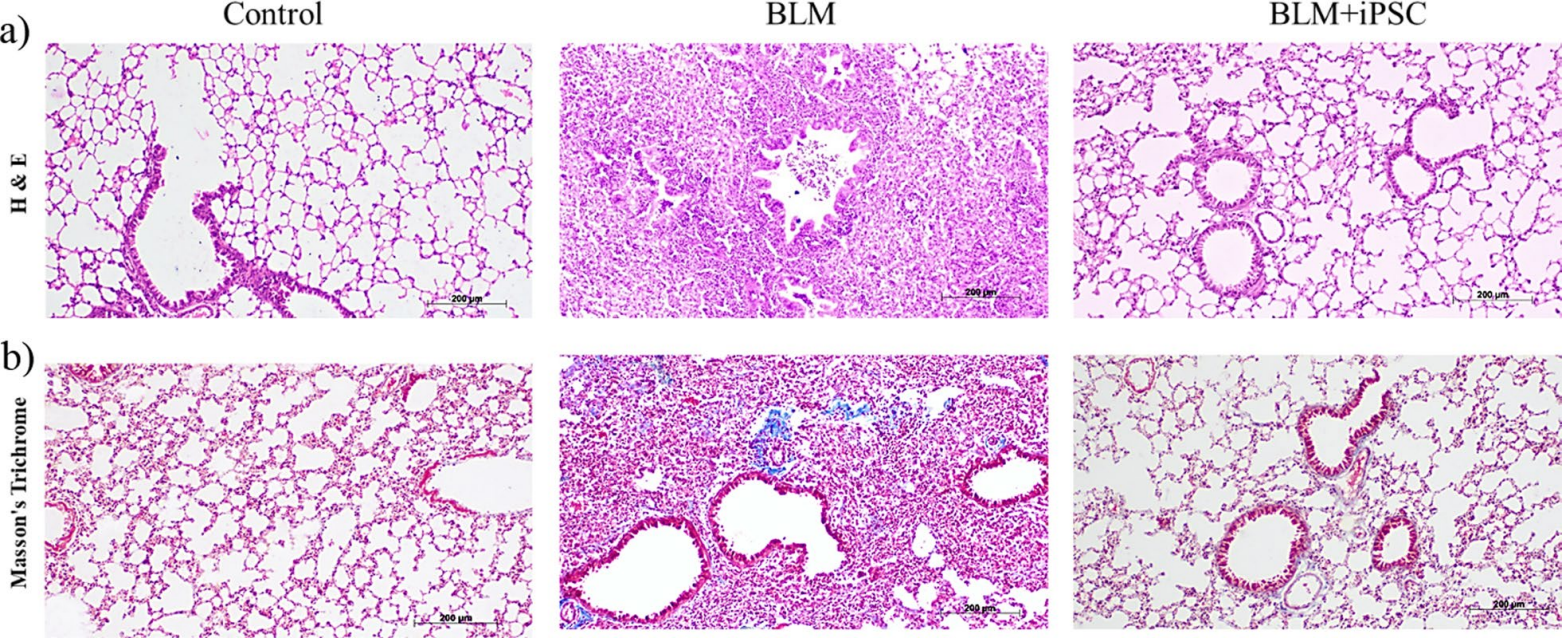
Histological assessment of lung fibrosis. **a** H&E staining of lung sections from control, BLM, and BLM + iPSC groups at 3 weeks after bleomycin administration. Scale bar = 100 μm. **b** Masson's trichrome staining of lung sections from control, BLM, and BLM + iPSC groups at 3 weeks after bleomycin administration. Scale bar = 200 μm

To confirm the establishment of the IPF model and to assess the antifibrotic effects of iPSCs by comparing the study groups, we performed histological analyses on lung tissues 3 weeks after bleomycin administration to evaluate the development and progression of lung fibrosis in the mouse model. We used hematoxylin and eosin (H&E) staining to assess the inflammation and alveolar damage and Masson's trichrome staining to quantify the collagen deposition and fibrosis. H&E staining revealed that bleomycin-induced severe inflammation and alveolar damage in the lung, characterized by infiltration of inflammatory cells, such as neutrophils, macrophages, and lymphocytes, thickening of alveolar septa due to fibroblast proliferation and collagen deposition, and formation of fibrotic foci consisting of myofibroblasts and extracellular matrix. In contrast, the control group showed normal lung architecture and minimal inflammation. The iPSC treatment significantly alleviated the inflammation and fibrotic lesions in the lung, as evidenced by reduced inflammatory cell infiltration, alveolar septal thickening, and fibrotic foci (Fig. 1A). Masson's trichrome staining confirmed that bleomycin-induced extensive collagen deposition and fibrosis in the lung, as indicated by the blue staining of collagen fibers. The iPSC treatment markedly reduced collagen deposition and fibrosis in the lung, as indicated by the decreased blue staining of collagen fibers (Fig. 1B). These results once more indicate that iPSCs can attenuate inflammation and fibrosis in the bleomycin-induced mouse model of IPF; They also suggest that iPSCs can improve the structure and function of the lung tissue by restoring the normal architecture and composition of the alveoli.

### Hydroxyproline assay of lung collagen content

**Fig. 2:**
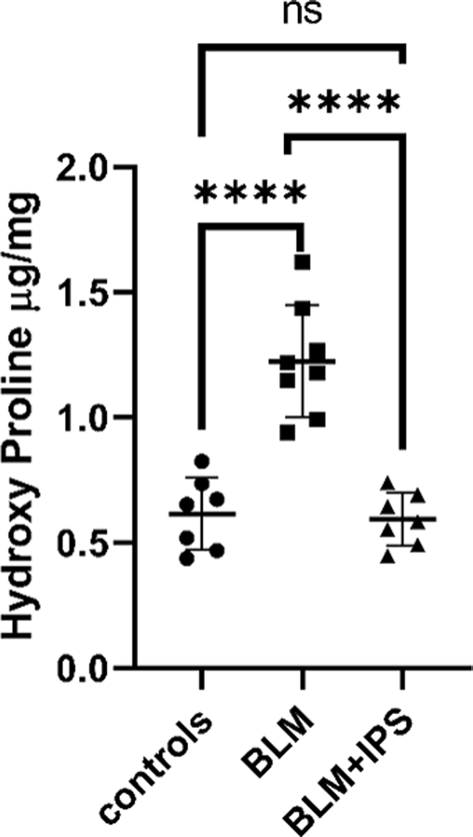
Hydroxyproline assay of lung collagen content. Hydroxyproline content in lung tissues from control, BLM, and BLM + iPSC groups at 3 weeks after bleomycin administration. The tissue used for hydroxyproline assay was the complete right lung. Data were normalized as per mg tissue; data were compared using the ANOVA method and presented as mean ± SD (n = 7 per group). ****P < 0.0001

To further confirm the establishment of the IPF mouse model and to assess the response to iPSC treatment in a quantitative manner, we performed a hydroxyproline assay on lung tissues 3 weeks after bleomycin administration. Hydroxyproline is a major component of collagen, and its content reflects the amount of collagen in the tissues. We found that bleomycin significantly increased the hydroxyproline content in the lung of the IPF model compared to the control group, indicating the accumulation of collagen and fibrosis. On the other hand, the iPSC treatment significantly decreased the hydroxyproline content in the lung of the treatment group (BLM + IPS) compared to the bleomycin group, indicating the reduction in collagen and fibrosis in the treated lung (Fig. 2). These results are consistent with the histological findings and further confirm the antifibrotic effects of iPSCs on IPF.

### Identification of iPSCs by real-time PCR

**Fig. 3:**
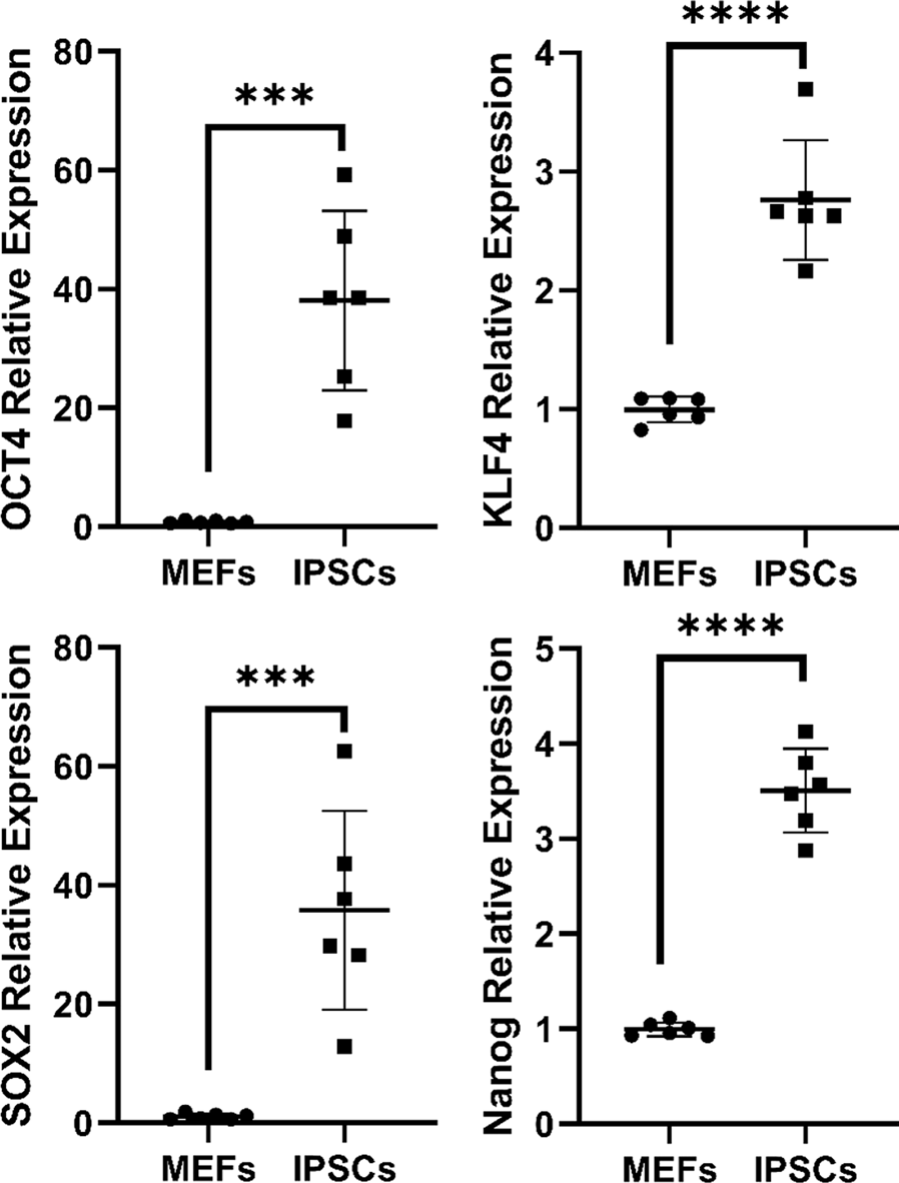
Identification of iPSCs by real-time PCR. Real-time PCR analysis of Oct4, Sox2, Klf4, and Nanog genes in iPSCs and MEFs. Data were analyzed using the t-test method and are presented as mean ± SD (n = 6 individual wells containing about 0.5 × 10^6 cells per group at passage 3). The iPSCs expressed significantly higher levels of Oct4, Sox2, Klf4, and Nanog genes than MEFs (***P < 0.001 vs. MEFs, ****P < 0.0001 vs. MEFs), indicating the successful reprogramming of somatic cells into pluripotent stem cells

After the successful establishment of the IPF model, we show that iPSCs were successfully generated from MEFs using a lentiviral vector with four pluripotency factors. To verify the identity and purity of the iPSCs, we measured the expression of pluripotency markers by real-time PCR. We collected RNA from iPSCs and MEFs after three passages and synthesized cDNA from them. Then, we compared the expression levels of the pluripotency markers between the two cell types. We measured the gene expression of four pluripotency markers: Oct4, Sox2, Klf, and Nanog. Oct4, Sox2, and Klf are transcription factors that are essential for maintaining the self-renewal and differentiation potential of pluripotent stem cells. Nanog is another transcription factor that is involved in the regulation of pluripotency and lineage specification. We found that iPSCs expressed significantly higher levels of Oct4, Sox2, Klf4, and Nanog genes than MEFs, indicating the successful reprogramming of somatic cells into pluripotent stem cells (Fig. 3). Interestingly, we also found that iPSCs expressed the Nanog gene even though it was not present in the original polycistronic cassette of the pluripotency factors in the lentivirus that was used for reprogramming. This suggests that the Nanog expression may be induced by other factors or mechanisms during the reprogramming process and further confirms the successful induction of the iPSCs.

### Gene expression analysis of Wnt signaling pathway

**Fig. 4:**
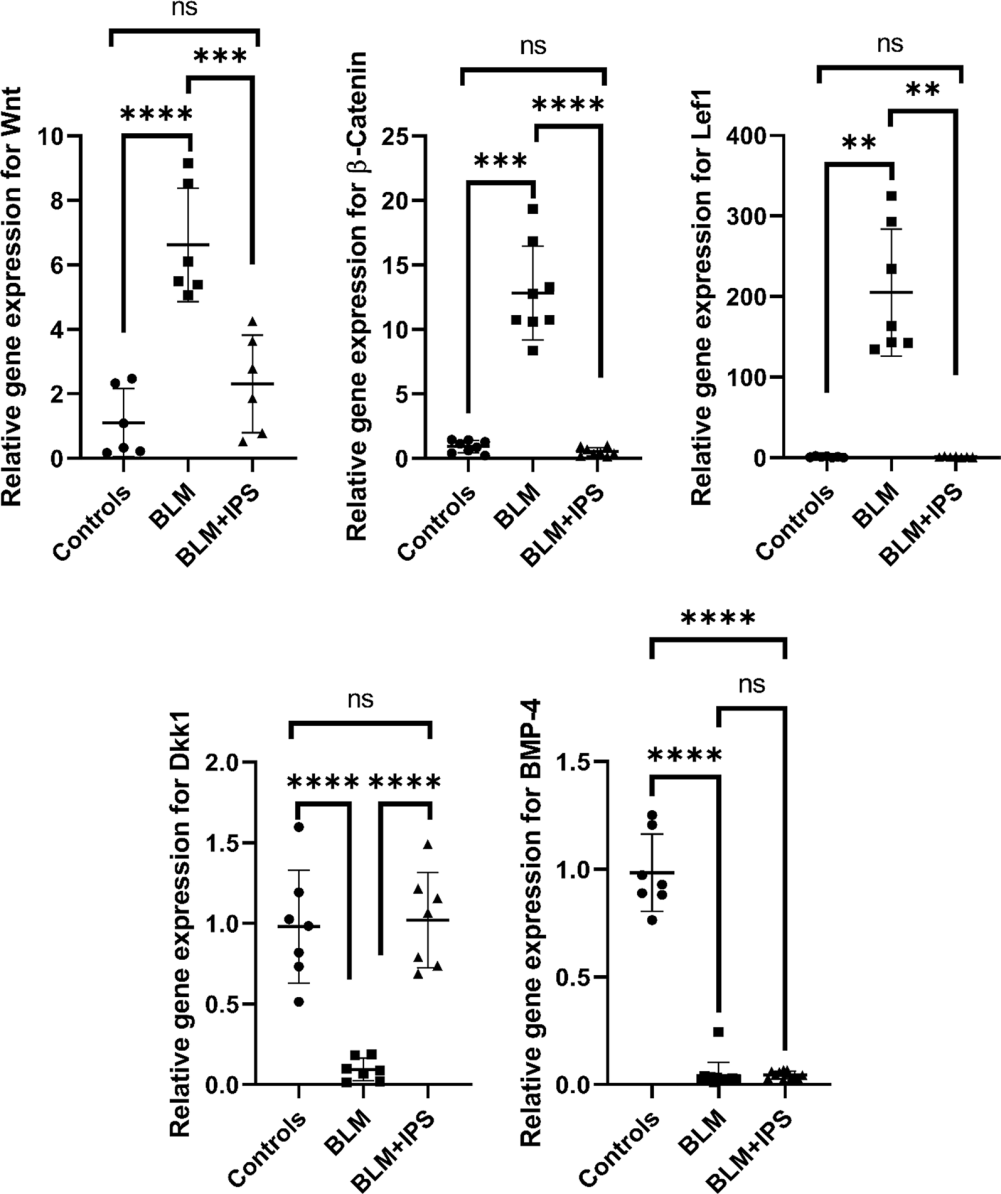
Gene expression analysis of Wnt signaling pathway. Real-time PCR analysis of Wnt, β-catenin, Lef, Dkk1, and Bmp4 genes in lung tissues from control, BLM, and BLM + iPSC groups at 3 weeks after bleomycin administration. Data were compared using the ANOVA method and are presented as mean ± SD. **P < 0.01, ***P < 0.001, ****P < 0.0001

After confirming the therapeutic effects of iPSCs on the IPF model according to the histological findings, we then aimed to investigate whether the iPSCs were capable of modulating the Wnt signaling pathway which is known to be implicated in IPF pathogenesis. As a result, we performed real-time PCR analysis on lung tissues 3 weeks after bleomycin administration. We measured the expression of Wnt-related genes (Wnt, β-catenin, Lef, and Dkk1, as well as the Bmp4 gene which is known to affect the Wnt pathway in several ways.) and compared them between iPSC-treated and control groups. WNT is a family of secreted glycoproteins that bind to cell surface receptors, such as Frizzled and LRP5/6, and activate downstream signaling cascades. β-catenin is a key mediator of canonical WNT signaling that translocates to the nucleus and interacts with LEF/TCF transcription factors to regulate gene expression. LEF/TCF is a family of transcription factors that bind to Wnt-responsive elements in the promoters of target genes and mediate Wnt-induced gene transcription. DKK1 is an inhibitor of Wnt signaling that binds to LRP5/6 receptors and prevents their interaction with Wnt ligands. We found that bleomycin significantly upregulated the expression of Wnt, β-catenin, and Lef genes in the lung compared to the control group, indicating the activation of the Wnt signaling pathway in IPF. In contrast, bleomycin significantly downregulated the expression of the Dkk1 gene, in the lung compared to the control group, indicating the suppression of the Wnt signaling pathway inhibitor in IPF. The iPSC treatment significantly reversed the changes in gene expression in the treated group compared to the bleomycin group, suggesting the modulation of the Wnt signaling pathway by iPSCs in IPF (Fig. 4). These results imply that iPSCs may exert their antifibrotic effects on IPF in part by restoring the balance of the WNT signaling pathway.

Moreover, we found that bleomycin administration downregulated the expression of the bone morphogenetic protein 4 (Bmp4) gene in the IPF lung tissue, while iPSCs treatment had no impact on the restoration of the Bmp4 expression. This also implies that iPSCs may inhibit WNT signaling by inducing Dkk1 expression independently of BMP4.

## Discussion

**Fig. 5:**
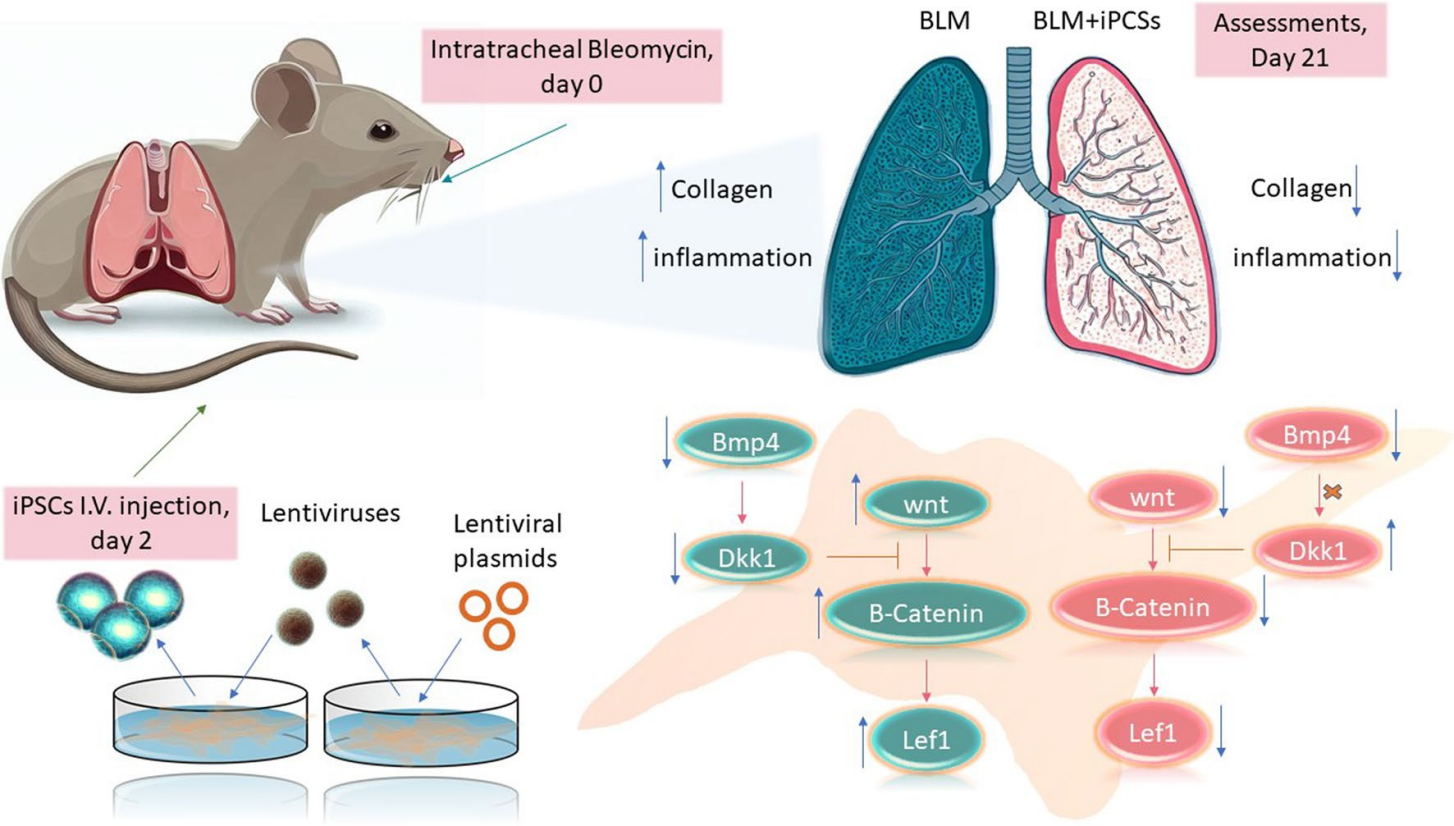
Schematic representation of the study flow. After successful induction of the iPSCs and the IPF mouse model, the iPSCs were injected via the tail vein, two days post bleomycin administration; on day 21, the histologic and molecular assessments were performed, and it was observed that besides alleviating the histologic features, iPSCs modulated the expression of the WNT pathway components which are known to contribute to the fibroblast transdifferentiation into myofibroblasts, although they exerted no effect on the reduced expression of the Bmp4 gene. BMP4 usually affects the WNT pathway by enhancing the expression of Dkk1

In this study, we evaluated the potential of iPSCs in modulating the expression of the key genes of the Wnt signaling pathway and BMP4 in a mouse model of IPF induced by bleomycin (Fig. 5). Herein, we used C57BL/6 mice as a multipurpose model for IPF research and chose the intratracheal route of Bleomycin administration as it produces a homogeneous distribution of fibrotic lesions among lung lobes. The rationale for using C57BL/6 mice is that they are susceptible to bleomycin-induced lung fibrosis, which is a widely used method to mimic IPF in animals [27–29].

We also confirmed the identity of iPSCs by real-time PCR for pluripotency markers. Despite the absence of the Nanog gene in the lentiviral construct that we employed to deliver the pluripotency factors, we found that iPSCs expressed the Nanog gene as well. This suggests that Nanog expression may be induced by other factors or mechanisms during the reprogramming process and may further confirm the successful induction of the iPSCs.

We administered iPSCs by tail vein injection 48 h after bleomycin instillation to evaluate their therapeutic effects on IPF. Subsequently, histological and molecular analyses were performed on lung tissues at 3 weeks after bleomycin administration; it was found that iPSCs significantly reduced the inflammation and fibrosis in the lung, as evidenced by H&E staining, Masson's trichrome staining, and hydroxyproline assay (Fig. 5). Consistently, several previous studies have reported the beneficial effects of IPSCs or their conditioned medium in animal models or cell cultures of pulmonary fibrosis. For instance, How et al. [30] showed that iPSC treatment mediated the release of interferon gamma-induced protein 10 (IP-10), in bronchoalveolar lavage fluid and serum from bleomycin-treated mice. Gazdhar et al. [31] have also demonstrated that the secretome of iPSCs (iPSC-Sec) treatment significantly improved lung function, and reduced lung inflammation and fibrosis, in bleomycin-treated mice; it was also shown that iPSC-Sec contained various growth factors and anti-inflammatory molecules. Furthermore, Zhou et al. [32], also have found that IPSCs suppressed inflammatory mediators involved in the initiation and progression of fibrosis, in lung tissues and fibroblasts from bleomycin-treated mice; also, they revealed that iPSC treatment suppressed the expression of pro-fibrotic factors and inhibited the activation of TGF-β1/Smad signaling. These studies suggest that IPSCs may exert their antifibrotic effects by modulating multiple molecular pathways that regulate fibrosis. However, the exact mechanisms and interactions of these pathways are still unclear and remain to be elucidated in future studies. Accordingly, our findings are in line with previous studies that reported the antifibrotic effects of iPSCs in IPF models. However, our study is novel in demonstrating that iPSCs can modulate the Wnt signaling pathway in IPF. The Wnt signaling pathway has been implicated in various aspects of IPF pathogenesis, such as epithelial-mesenchymal transition (EMT), fibroblast activation and differentiation into myofibroblasts, ECM remodeling, angiogenesis, inflammation, and apoptosis [33, 34].

We also assessed the expression of Wnt signaling pathway genes (Wnt, β-catenin, Lef, and Dkk1); it was subsequently found that Wnt, β-catenin, and Lef genes were upregulated while Dkk1 was downregulated in the bleomycin-induced IPF model and iPSCs treatment normalized their expression. These results indicate that iPSCs may modulate the Wnt signaling pathway in IPF by downregulating the expression of Wnt activators and upregulating the expression of Wnt inhibitors. In the current study, we have also shown that bleomycin treatment would downregulate the expression of the BMP4 gene which is consistent with the concurrent downregulation of the Dkk1 gene and previous data [35], although the treatment of the IPF mice with iPSCs did not affect restoring the expression of BMP4 gene. This could indicate that BMP4 may not be essential for the regulation of WNT signaling by iPSCs in IPF which could be explained by several possibilities: First, iPSCs may secrete other factors that can inhibit WNT signaling [36]. Second, iPSCs may alter the expression or activity of other components of the WNT signaling pathway, such as LRP5/6 or GSK3β [6]. Third, iPSCs may affect the cross-talk between WNT signaling and other pathways that are involved in IPF pathogenesis, such as transforming growth factor-β (TGF-β) [35]. Overall, our findings could provide new insights into the molecular mechanisms and therapeutic potential of iPSCs for IPF treatment.

Modulating the Wnt signaling pathway may have multiple beneficial effects on IPF which is further supported by previous studies similar to our study but different in some aspects such as the disease model, the intervention, or outcome measures. For instance, Konigshoff et al. [37] found that the expression of Wnt ligands and receptors, as well as β-catenin was increased in lung tissues from IPF patients; it was also shown that Wnt ligands stimulated the proliferation and collagen production of lung fibroblasts, which is consistent with our findings that Wnt signaling pathway genes were upregulated in the bleomycin-induced IPF model and iPSCs treatment normalized their expression. However, this study did not use iPSCs or any other stem cells as a therapeutic intervention, nor did it measure markers of inflammation and fibrosis in the lung. Besides, Several similar studies have addressed the potential of treatment of pulmonary fibrosis in different murine models by inhibiting the WNT pathway using various therapeutic compounds such as dehydrozingerone (DHZ), a natural compound derived from ginger, XAV-939, and ICG-001 which are small molecule inhibitors of the WNT/β-Catenin pathway; all of which have shown promising results in attenuating lung inflammation and fibrosis [38–41]. These data are in line with our results highlighting the importance of the WNT pathway in pulmonary fibrosis and supporting the theory of targeting it as a possible therapeutic approach. However, utilizing cell therapy would have multiple advantages over these compounds as it may provide a source of alveolar epithelial cells or other lung cell types that can replace the damaged or lost cells and restore the lung function and structure [42, 43]. Also, Cell therapy may provide anti-inflammatory and antifibrotic factors [30–32] and may modulate multiple molecular pathways [42–44].

In a recent 2023 study similar to ours, the effects of umbilical cord mesenchymal stem cells (UCMSCs) were investigated on acute respiratory distress syndrome (ARDS)-associated pulmonary fibrosis in mice; the authors have found that UCMSCs significantly improved lung function, and reduced inflammation and fibrosis, by modulating the Wnt/β-catenin pathway [42]. However, this study used a different type of stem cells, a different disease model, and different outcome measures (lung function) than our study. Furthermore, in 2018, another relevant study has shown that bone marrow mesenchymal stromal cells (BMSCs) significantly improved lung function, reduced lung inflammation and fibrosis, and increased survival rate in silica-exposed rats by attenuating the activation of Wnt/β-catenin signaling, and restoring the expression of Wnt inhibitors and antagonists, such as DKK1 [43]. Although these two individual studies are in line with our data showing the possible effects of cell therapy on pulmonary fibrosis, the bleomycin-induced model utilized in our study better recapitulates the human IPF, which is the focus of our study; also, iPSCs have multiple benefits over the MSCs, such as unlimited availability and accessibility, as they can be derived from various somatic cells of any individual [45]. Besides, iPSCs have high quality and potency, with equal and controlled effects, unlike their MSC counterparts with varying potencies and different inflammatory/anti-inflammatory properties [45]. Quite contrary to our data one study in 2023 found that DKK1 expression was increased in lung tissues and blood samples from patients with IPF, as well as in mice treated with bleomycin. It was also shown that DKK1 promoted fibroblast activation and differentiation, by suppressing the WNT/β-Catenin pathway and enhancing the TGF-β/Smad pathway [46]. This discrepancy may be due to different experimental conditions, such as the dose and time point of bleomycin administration, or the method of measuring DKK1 expression. Alternatively, it may reflect the complexity and diversity of the Wnt signaling pathway in different conditions.

These studies suggest that targeting the Wnt signaling pathway could be a viable therapeutic strategy for pulmonary fibrosis. However, further research is needed to fully understand the mechanisms of action of these compounds and to evaluate their safety and efficacy in human clinical trials. Additionally, pulmonary fibrosis is a complex disease with multiple contributing factors, so a combination of therapeutic approaches may be necessary for effective treatment.

Lung function data, such as forced vital capacity (FVC) and forced expiratory volume in 1 s (FEV1), can reflect the degree of lung fibrosis and inflammation in BLM-induced IPF mice, as well as the response to iPSC treatment. Lung function data are also easier and faster to measure than histological or molecular data, which require invasive procedures and sophisticated techniques. Therefore, lung function data may be more relevant and meaningful for assessing the therapeutic effect of iPSCs on IPF in BLM-induced IPF mice, as they can directly evaluate the functional improvement or deterioration of the lungs after iPSC injection.

The findings of our study and the previous studies that have been discussed so far are very promising for the treatment of IPF in animal models, but they need to be validated and translated to human patients with IPF. There are currently no clinical trials involving iPSCs for IPF in humans [47]. However, there are some ongoing or planned clinical trials using iPSCs for other diseases, such as macular degeneration, Parkinson's disease, spinal cord injury, and heart failure [48]. These trials may provide valuable insights and experience for the future application of iPSCs for IPF in humans. Some of the challenges and barriers that need to be overcome include the safety and efficacy of iPSCs, the immune compatibility of iPSCs, the ethical and regulatory issues of iPSCs, and the cost and scalability of iPSCs [47]. There are several ways to improve the safety and efficacy of iPSCs, such as: Using non-integrative methods to generate iPSCs, which avoid the risk of insertional mutagenesis and genomic instability caused by viral vectors, or using xeno-free and feeder-free culture conditions to maintain iPSCs, which reduce the risk of contamination and immunogenicity from animal-derived products [47, 48]. These methods may help to improve the safety and efficacy of iPSCs for various therapeutic applications. However, more research and optimization are needed to overcome the remaining challenges and limitations.

## Limitations

Our study has some limitations that need to be addressed. First, the bleomycin-induced mouse model of IPF may not fully recapitulate the human disease, which has a more complex and heterogeneous etiology and pathogenesis. Therefore, the results obtained in this model may not be directly applicable to human patients with IPF. Second, the iPSCs used in this study were generated from mouse embryonic fibroblasts, which may have different characteristics and properties from human iPSCs derived from adult somatic cells. Therefore, the safety and efficacy of iPSCs in human IPF may differ from those observed in mice. Third, the dose and timing of iPSC administration may affect the outcome of the treatment. In this study, we used a single dose of 1 × 10^6 iPSCs delivered by tail vein injection 48 h after bleomycin instillation. However, other doses, routes, or schedules of iPSC administration may have different effects on lung fibrosis and inflammation. Therefore, further optimization and comparison of different iPSC delivery methods are needed to determine the optimal therapeutic regimen. Fourth, the mechanism of action of iPSCs in modulating the Wnt signaling pathway in IPF is not fully elucidated in this study. We only measured the expression of four prominent genes (Wnt, β-catenin, LEF, and DKK1) involved in the Wnt signaling pathway as well as the BMP4 gene by real-time PCR. However, many other components and regulators of the Wnt signaling pathway may also play a role in IPF pathogenesis and iPSC treatment. Therefore, more comprehensive and in-depth analyses of the Wnt signaling pathway at the protein and functional levels are needed to clarify the mechanism of action of iPSCs in IPF. Also, we did not test functional readouts to show pluripotency, such as teratoma formation or differentiation into three germ layers, because we were mainly interested in the immunomodulatory and regenerative effects of iPSCs on lung fibrosis. This may limit the generalizability of our findings to other applications of iPSCs, such as cell replacement therapy or disease modeling. Likewise, we did not perform bronchoalveolar lavage (BAL) on our mice, because we wanted to preserve the lung tissue for histological and molecular analyses. However, BAL cells would provide valuable information about the immune status and cytokine profile of the lungs after iPSC treatment. We did not acquire any lung function data, such as spirometry or plethysmography, because we did not have access to these equipment or techniques in our laboratory. Lung function data would be more relevant and meaningful than histological or molecular data for assessing the therapeutic effect of iPSCs on IPF.

We did not measure the animal’s food and water intake in our study. We acknowledge that this could be a potential confounder for the data interpretation, as reduced food intake, and dehydration are common side effects of bleomycin administration in mice, and they may reflect the severity of lung injury and may influence the tissue repair. Therefore, future studies should include these parameters as part of the experimental design and analysis.

Measuring proximate blockers of OSKM, such as TSP1 and its receptors in the lung tissue before and after iPSC injection, could be a potential mechanism to explore in future studies, as it could provide some insights into how iPSCs affect the fibrotic process and the lung microenvironment. TSP1 is a matricellular protein that has pro-fibrotic functions and may interfere with the reprogramming and regeneration of lung tissue by iPSCs [49–51]. TSP1 also modulates nitric oxide, activates latent TGFβ1 and binds to receptors CD36 and CD47, which may affect the cell metabolism and survival of iPSCs [52]. Therefore, by measuring the levels of TSP1, we could assess the impact of iPSCs on the expression and signaling of these molecules, and how they modulate the fibrotic response.

## Conclusion

In conclusion, we have demonstrated that iPSCs can attenuate inflammation and fibrosis in the bleomycin-induced mouse model of IPF. We have also shown that iPSCs can modulate the aberrantly expressed genes of the Wnt signaling pathway, which is a key regulator of fibrogenesis, by restoring the balance of its activators and inhibitors. These results suggest that iPSCs may have therapeutic potential for IPF and other fibrotic diseases. However, there are still some limitations and challenges that need to be addressed before iPSCs can be translated into clinical applications. For instance, the safety and efficacy of iPSCs, the optimal dose and route of administration, and the molecular mechanisms of iPSCs in modulating other signaling pathways and cell types involved in IPF need to be elucidated. Therefore, we propose that future research should focus on these aspects and explore the possibility of combining iPSCs with other strategies, such as gene therapy, drug delivery, or tissue engineering, to enhance their antifibrotic effects and overcome their limitations."

## Data Availability

The data are available on reasonable request.

## Abbreviations

IPS: Induced pluripotent stem cells
BLM: Bleomycin
PBS: Phosphate-buffered saline
H&E: Hematoxylin and eosin
SYBR: SYBR Green I
IPF: Idiopathic pulmonary fibrosis
WNT: Wingless/Integrated
BMP4: Bone morphogenetic protein 4
iPSCs: Induced pluripotent stem cells
ECM: Extracellular matrix
TGF-β: Transforming growth factor beta
CTGF: Connective tissue growth factor
EMT: Epithelial-mesenchymal transition
DKK1: Dickkopf-1
FRPs: Secreted frizzle-related proteins
WIF-1: Wnt inhibitory factor-1
Smad: Sma and Mad-related proteins
YAP/TAZ: Yes-associated protein/transcriptional coactivator with PDZ-binding motif
IL-10: Interleukin 10
VEGF: Vascular endothelial growth factor
AECs: Alveolar epithelial cells
ECs: Endothelial cells
LRP5/6: Low-density lipoprotein receptor-related protein 5/6
LEF/TCF: Lymphoid enhancer factor/T-cell factor
TSP1: Thrombospondin-1
